# SPECIES: A platform for the exploration of ecological data

**DOI:** 10.1002/ece3.4800

**Published:** 2019-01-28

**Authors:** Christopher R. Stephens, Raúl Sierra‐Alcocer, Constantino González‐Salazar, Juan M. Barrios, Juan Carlos Salazar Carrillo, Everardo Robredo Ezquivelzeta, Enrique del Callejo Canal

**Affiliations:** ^1^ Centro de Ciencias de la Complejidad Universidad Nacional Autónoma de México Mexico City Mexico; ^2^ Instituto de Ciencias Nucleares Universidad Nacional Autónoma de México Mexico City Mexico; ^3^ Comisión Nacional para el Conocimiento y Uso de la Biodiversidad (CONABIO) Mexico City Mexico; ^4^ Departamento de Ciencias Ambientales Universidad Autónoma Metropolitana Unidad Lerma Estado de Mexico Mexico

**Keywords:** data mining, ecology, networks, niche modeling

## Abstract

The modeling of ecological data that include both abiotic and biotic factors is fundamental to our understanding of ecosystems. Repositories of biodiversity data, such as GBIF, iDigBio, Atlas of Living Australia, and SNIB (Mexico's National System of Biodiversity Information), contain a great deal of information that can lead to knowledge discovery about ecosystems. However, there is a lack of tools with which to efficiently extract such knowledge. In this paper, we present SPECIES, an open, web‐based platform designed to extract implicit information contained in large scale sets of ecological data. SPECIES is based on a tested methodology, wherein the correlations of variables of arbitrary type and spatial resolution, both biotic and abiotic, discrete and continuous, may be explored from both niche and network perspectives. In distinction to other modeling systems, SPECIES is a full stack exploratory tool that integrates the three basic components: data (which is incrementally growing), a statistical modeling and analysis engine, and an interactive visualization front end. Combined, these components provide a powerful tool that may guide ecologists toward new insights. SPECIES is optimized to support fast hypothesis prototyping and testing, analyzing thousands of biotic and abiotic variables, and presenting descriptive results to the user at different levels of detail. SPECIES is an open‐access platform available online (http://species.conabio.gob.mx), that is, powerful, flexible, and easy to use. It allows for the exploration and incorporation of ecological data and its subsequent integration into predictive models for both potential ecological niche and geographic distribution. It also provides an ecosystemic, network‐based analysis that may guide the researcher in identifying relations between different biota, such as the relation between disease vectors and potential disease hosts.

## 1 INTRODUCTION

A fundamental goal of ecology is to understand, model, and quantify ecological associations, some of which may represent ecological interactions, both biotic and abiotic, at different geographical and temporal scales (Kissling et al., 2012; Wisz et al., 2013). Two important, associated conceptual frameworks are that of niche and community, where both biotic and abiotic factors play important roles, for instance, in defining the niche of a taxon (Soberón, 2007) and its geographic distribution. However, it has been a challenge to incorporate both types of factor into distribution modeling (Araújo & Luoto, 2007; Kissling et al., 2012). Most progress has been made using environmental layers and different statistical models (Qiao, Soberón, & Peterson, 2015; Vaz, Cunha, & Nabout, 2015) to map the relationship between them and an output variable, such as the propensity for the presence of a given species. In terms of community, one of the most popular representations is as a network, such as a food web (Pimm, Lawton, & Cohen, 1991). Such networks normally represent only known direct ecological interactions (Vázquez, Morris, & Jordano, 2005), such as predation (Ives & Carpenter, 2007), as opposed to more indirect interactions, such as facilitation (Valiente‐Banuet & Verdú, 2007). SPECIES uses both perspectives, the niche and network perspectives, to explore associations found in the data between groups of species and/or abiotic spatial features. In this way, the analyst can focus on any subset of potential associations of interest to examine which ones have a ready known or plausible ecological interpretation and also what that interpretation may be.

In addition to the complexity of characterizing or classifying associations, such as interactions, the degree of multifactoriality in ecological systems is such that the number of actual or potential associations and interactions is much greater than the number of observed, or observable, ones. Even under the assumption that all potential pairwise interactions were observable, the number of associations that we would have to check grows quadratically with the number of species present in an area. For example, in the SNIB database, if we use cells with 8 km per side, we obtain some cells with the presence of more than a thousand animal species. If we add plants, then we get cells with more than 3,000 species. That is, we would have to study, respectively, more than 499,500 and 4,498,500 associations, some of which do, or could, represent pairwise interactions. Of course, no one would attempt such an enterprise, as biological knowledge and observation are important guides with which to look for specific interactions. However, as we do not have an a priori understanding of all the potential biotic relations that are worth investigating, the question then arises: Can we reduce the search space using proxy data, such as occurrence data? The general approach of using occurrence data to potentially characterize ecological interactions has a long history and is still a theme of debate (Bell, 2005; Connor & Simberloff, 1979, 1983; Diamond, ; Gilpin & Diamond, 1982; Hubbell, ; Simberloff & Connor, 1984; Ulrich, Almeida‐Neto, & Gotelli, 2009; Ulrich et al., 2017). In the case of biotic factors, its quantification has been chiefly associated with the analysis of presence–absence matrices (Arita, Christen, Rodríguez, & Soberón, 2012; Gaston, Chown, & Evans, 2008). In the case of abiotic interactions, standard distribution modeling (Araújo & Guisan, 2006; Rangel & Loyola, 2012) is an attempt to do this. However, a theoretical and computational framework in which both biotic and abiotic factors of arbitrary spatial resolution may be integrated at both the niche and community levels has been, until recently, lacking.

SPECIES is a software platform for doing just that. It is an exploratory tool designed to help in the construction of ecological hypotheses. It is meant to be part of a researcher toolset that opens access to information hidden in large databases like SNIB or GBIF. Although such historical data suffer from several problems, in particular data bias due to incomplete observation efforts, it is undeniable that such databases also contain valuable information that has been assembled with great effort by many institutions and individuals, and which could lead to new insights and results in ecology and related fields. SPECIES can quickly process millions of records for thousands of species in order to detect and quantify correlations between biotic or abiotic features represented by spatial distributions of arbitrary resolution. Of course, correlation is not causation and, as is well known, such geographic correlations must be interpreted by a specialist in the context of the question being asked (the selection of groups of species and abiotic factors) and the specialist knowledge.

For a deeper discussion of its underlying theoretical framework, we refer the reader to (González‐Salazar, Stephens, & Marquet, 2013; Sierra & Stephens, 2012; Stephens et al., 2009). This framework has been applied to various systems, principally in the prediction of host–vector interactions in zoonoses (González‐Salazar & Stephens, 2012; González‐Salazar et al., 2013; Rengifo‐Correa, Stephens, Morrone, Téllez‐Rendón, & González‐Salazar, 2017; Stephens et al., 2009), where the predictions have been validated by empirical observation (Berzunza‐Cruz et al., 2015; Stephens et al., 2016).

## 2 MATERIALS AND METHODS

The SPECIES platform takes as input any spatio‐temporal variable $\hat{X}$ defined over a region R and time interval T, where, for example, $\hat{X}$ could be the presence of a specific organism, or a real‐valued variable such as average annual temperature. Partitioning R into a grid, G with *N* cells, we obtain a collection of *N* sites, $c_{i}$, i=1,…,N. In the current version, SPECIES 1.0, we use uniform, rectangular grids of 8, 16, 32, and 64 km per cell side. For Boolean spatial variables, the mapping from R to G is straightforward: Let $c_{i}$ be a site, we then define X such that X=1 if $c_{i}$ contains a point where $\hat{X}=1$ and zero otherwise. To treat all variables on the same footing, we transform any non‐Boolean variable into a discrete set of Boolean variables by dividing its range into a number of discrete intervals. In the current version, each such variable is divided into 10 such intervals (deciles). Importantly, it is this mapping which brings all variables to the same spatial resolution, as defined by G. For example, if we have a real‐valued variable $\hat{X}$, such as “Annual Mean Temperature,” since in SPECIES 1.0, the principal mapping is “presence/no presence”,^1^ $\hat{X}$ is discretized by splitting it into ten “presence/no presence” variables $X_{1},\cdots X_{10}$. This means that for an environmental layer, if there are pixels in a cell (site) corresponding to a particular range/bin, then the cell contains a “presence” of that variable range. Analogously, we could extend this logic from a spatial region to a spatio‐temporal region, R×T. Note that, although converting all variables to Boolean type allows for a fair comparison of all variables, independent of their spatial resolution, in the discretization process, some information may be lost due to the coarse graining. This can, of course, be reduced by considering more bins, as long as in each bin there are enough data points. The result of this process can be easily conceptualized as a big presence–absence matrix (PAM), a familiar analytical tool in macroecology and biogeography (Arita et al., 2012; McCoy & Heck, 1987). The distinction with standard PAMs (which are species × locations matrices) is that in SPECIES, rows represent species and any other type of “presence/no presence” variable, and columns are grid cells.

Let *X* and *Y* be two Boolean variables, then the degree of co‐occurrence between them is measured with respect to the null hypothesis that *X* is independent of *Y*, that is *P*(*X*|*Y*) = *P*(*X*). As we do not know the underlying probability distributions, we must estimate them from data. For example, $\hat{P}\left(X\right)=N_{X}/N$, where *N*_*X*_ is the number of cells with the presence of feature *X*, and *N* is the total number of grid cells. Note that, when the Boolean variable of interest is a species presence, we do not assume that $\hat{P}\left(X\right)=N_{X}/N$ is an unbiased estimator of the real number of counts of presences in any cell, as would be found from a systematic sampling and documentation of the range of species. In this case, in general, $\hat{P}\left(X\right)=N_{X}/N$ would be an underestimate, due to incomplete observation efforts (Bojorquez‐Tapia, Azuara, Ezcurra, & Flores‐Villela, 1995; Soberón, 2007; Soberón, Llorente, & Oñate, 2000; Yesson et al., 2007). To test the dependence of *X* and *Y* relative to the null hypothesis, we calculate the estimator, $\hat{P}\left(X|Y\right)=N_{X,Y}/N_{Y}$, of the conditional probability P(X|Y), where *N*_*X,Y*_ is the number of cells with *X* = 1 and *Y* = 1, that is, where the spatial features the variables represent co‐occur. Once again, we do not assume that, in the case of species presence, $\hat{P}\left(X|Y\right)=N_{X,Y}/N_{Y}$ is an unbiased estimator of the real number of co‐occurrences of presences in any cell.

As the null hypothesis corresponds to a binomial process with probability $\hat{P}\left(X\right)$, we may naturally use a binomial test to determine the statistical significance of the degree of co‐occurrence using standard hypothesis testing. We denote the corresponding test statistic as ε. Explicitly

(1) $$\begin{array}{cc} \varepsilon (X|Y) & =\frac{N_{Y}\left(\hat{P}\left(X|Y\right)-\hat{P}\left(X\right)\right)}{\sqrt{N_{Y}\hat{P}\left(X\right)\left(1-\hat{P}\left(X\right)\right)}}\\ & =\frac{N_{Y}\left(\frac{N_{X,Y}}{N_{Y}}-\frac{N_{X}}{N}\right)}{\sqrt{N_{Y}\left(\frac{N_{X}}{N}\right)\left(1-\frac{N_{X}}{N}\right)}}\\ & =\frac{N_{X,Y}-N_{Y}\frac{N_{X}}{N}}{\sqrt{N_{Y}\left(\frac{N_{X}}{N}\right)\left(1-\frac{N_{X}}{N}\right)}}. \end{array}$$

The conditional probability estimators $\hat{P}\left(X|Y\right)$ and $\hat{P}\left(Y|X\right)$, and the statistical diagnostic ε(X|Y), are the base building blocks from which species distributions, species niches, and communities can be constructed. The statistic ε(X|Y) measures the degree of statistical significance of the co‐occurrence between *X* and *Y*. Although the existence of a significant co‐occurrence is not a sufficient condition for the existence of an interaction between *X* and *Y*, it is a necessary condition, and therefore can serve as a filter on the space of associations to indicate from which subset biotic interactions are most likely to be found. Thus, potential biotic interactions may be explored in SPECIES, though a confirmation of the interaction requires data other than co‐occurrence data. A ranking of species as covariates in terms of ε(X|Y), for example, has been used to identify disease hosts (González‐Salazar & Stephens, 2012; González‐Salazar et al., 2013; Rengifo‐Correa et al., 2017; Stephens et al., 2009, 2016). In Section 3.1, as use cases, we show how it is consistent with identifying known food sources for two species: *Panthera onca* and *Baronia brevicaunis*.

### 2.1 Niche and community inference

We take as a mathematical representation of the niche for a taxon, C, the probability function P(C|X), where X represents the set of potential niche variables under consideration—both abiotic and biotic—present in a given site. In SPECIES 1.0, the posterior probability P(C|X) is calculated using Bayes’ theorem and the Naïve Bayes’ approximation (NBA; Stephens, Huerta, & Linares, 2017). In principle, other models could be used, such as MaxEnt, or logistic regression. We use the Naïve Bayes approximation for its transparency and ease, and speed of calculation.

Usually, rather than a direct estimation of P(C|X), the score function

(2) $$S\left(C|X\right)=\ln\left(\frac{\hat{P}\left(C|X\right)}{\hat{P}\left(\overset{¯}{C}|X\right)}\right),$$

is used, where $\overset{¯}{C}$ is the set complement of *C*, that is, in the present case those cells where there is no presence of *C*. Using Bayes theorem and the Naïve Bayes approximation for the likelihood, $P\left(X|C\right)=\prod_{i=1}^{N}P\left(X_{i}|C\right)$, we have

(3) $$\begin{array}{cc} S(C|X) & =\sum_{i=1}\ln\left(\frac{\hat{P}\left(X_{i}|C\right)}{\hat{P}\left(X_{i}|\overset{¯}{C}\right)}\right)+\ln\left(\frac{\hat{P}\left(C\right)}{\hat{P}\left(\overset{¯}{C}\right)}\right)\\ & =\sum_{i=1}\ln\left(\frac{\frac{N_{C,X_{i}}}{N_{C}}}{\frac{N_{X_{i}}-N_{C,X_{i}}}{N-N_{c}}}\right)+\ln\left(\frac{N_{C}}{N-N_{C}}\right)\\ & =\sum_{i=1}\ln\left(\frac{N_{C,X_{i}}\left(N-N_{C}\right)}{N_{C}\left(N_{X_{i}}-N_{C,X_{i}}\right)}\right)+\ln\left(\frac{N_{C}}{N-N_{c}}\right). \end{array}$$

In SPECIES 1.0, biotic niche variables are considered to be presence/no presence, so that $\hat{P}\left(X_{i}|C\right)=N_{C,X_{i}}/N_{C}$, where $N_{C,X_{i}}$ is the number of cells with presence of the target taxon/group C and presence of the niche variable *X*_*i*_. Similarly, $\hat{P}\left(X_{i}|\overset{¯}{C}\right)=N_{\overset{¯}{C},X_{i}}/N_{\overset{¯}{C}}$, where $N_{\overset{¯}{C},X_{i}}$ is the number of cells with presence of the niche variable *X*_*i*_ but no presence of the taxon/group C. When $N_{C,X_{i}}=0$ or $N_{C,X_{i}}=N_{X_{i}}$, leading to $\hat{P}\left(X_{i}|C\right)=0$ or $\hat{P}\left(X_{i}|\overset{¯}{C}\right)=0$, some degree of smoothing must be implemented. Currently, we use a standard Laplace correction.

Note that by converting all variables, both abiotic and biotic, to one or more Boolean variables we can, using the Naive Bayes approximation, compare the contributions of any variables to the presence of any given species. Thus, we may, for example, consider if the presence of a given prey species for a predator is associated with a greater score than a given annual temperature range. A high score for a given variable, *X*_*i*_, indicates that the variable discriminates well between presence and no presence of the target species. However, it does not tell us “why” it has a high score. Such a high score may be consistent with current knowledge; for example, a high score associated with a known prey species of a given predator; or it may be indicative of a potential interaction, for example, a prey species that has not been observed as such. This latter is purely an inference, however, and must be confirmed by a suitable experiment.

Another advantage of the Naïve Bayes approximation is that it allows for a simple evaluation of the impact of variable aggregation. For instance, it is possible to determine the relative contribution as niche variables of bats versus rodents, or biotic versus abiotic variables as a whole. The overall contribution of a group of variables depends on both the predictability inherent in each factor and the number of factors in the group. The platform uses fivefold cross‐validation to generate model evaluation metrics. We use recall/sensitivity^2^ as the principle performance measure.

Often the Naïve Bayes approximation is itself naively criticized due to its independence assumption. However, it remains as one of the most universally used (across multiple problem areas) machine learning algorithms. Despite its simplicity and its apparently strong assumption of independence between variables, it has proved to be a very robust classifier, even in problems, such as text analysis, where it is known that there are strong correlations between features. Indeed, the NBA can be very competitive with respect to many other seemingly more advanced techniques in terms of performance, say, considering the area under the ROC curve (Zhang & Su, 2008). There have been several papers explaining why it works so well, and this has been analyzed in detail recently, where it was shown that it is inevitable that the signed deviations from independence can largely cancel out between different feature combinations thus leading to the robust performance of the algorithm as being an emergent property (Stephens, Huerta, et al., 2017).

In summary, SPECIES allows for the construction of species distribution models using both biotic and abiotic drivers of arbitrary spatial resolution, which can be compared both in terms of their magnitude and sign. However, the precise ecological interpretation of the contribution of a given niche variable requires further knowledge. This is the case for both abiotic and biotic drivers.

The basic hypothesis that co‐occurrence associations can be used to explore possible ecological interactions is also applicable at the community level, where we consider multiple taxa/groups, forming a network whose nodes are defined by any group labels, and whose weighted links are defined using a statistical co‐occurrence measure, such as ε. SPECIES allows the user to select groups of features of interest, which can be made of biotic or abiotic variables, or to filter associations according to their statistical significance.

## 3 SYSTEM DESIGN

Many ecological modeling applications consist of a downloadable software that is used to analyze a proprietary dataset. In contrast, SPECIES uses a web‐based application that is directly tied to large, public datasets, thereby permitting analyses that would be impossible with smaller, bespoke datasets. SPECIES 1.0 has been constructed on top of the National Biodiversity Information System (SNIB for its initials in Spanish), “the largest and most complete database on the biodiversity of any country (9.6 million specimens, all taxonomically vouched by specialists and accurately georeferenced) housed under a single institutional roof” (Sarukhán & Jiménez, 2016), and is currently being extended to consume data from the Global Biodiversity Information Facility (GBIF: The Global Biodiversity Information Facility, 2017). There are two principle routes for system/user interactions: The first is a web GUI, focusing on providing quick hypothesis building and testing, and with an emphasis on exploratory analysis. The second is a web API, which supports users that may want to use it with other platforms, such as R or Python. SPECIES also provides tools to share the results of an analysis: Tables can be exported in CSV or Excel format; maps can be downloaded as GeoJSON; and ecological networks can be downloaded as CSV files. Another way to share an analysis is by sharing the setup of the analysis via a URL that reproduces the exact setup of the experiment.

### 3.1 Data sources

SPECIES 1.0 currently integrates three distinct data types: geographical points (such as species observations); raster data (such as climate layers, topographic layers); and geographical partitions (squares, Census blocks, etc.), which are used to determine co‐occurrences. Currently, the data sources on SPECIES 1.0 are as follows: SNIB's point‐collection database, WorldClim 1.4 variables for Mexico, and grids for Mexico, at cell sizes of 8, 16, 32, and 64 km per side. Also available is a development version of SPECIES, accessible at species.conabio.gob.mx/dev/, that in addition to the data just mentioned also includes US‐GBIF observations from the class Mammalia and the family Reduviidae (GBIF.org, 2017a, 2017b), extended sections of WorldClim 1.4 variables that include Mexico and the United States, excluding Alaska and Hawaii; and extended grids to cover the aforementioned area. The number of species and occurrences included in SPECIES 1.0 and the development version, respectively, can be seen in Table 1, while the number of bioclimatic variables and the number of grid cells by resolution for each version can be seen in Tables 2 and 3, respectively.

**Table 1:** Species data integrated into the SPECIES database

Source	No. of species	No. of occurrences
CONABIO—SNIB	81,603	8,811,744
GBIF—US^a^	4,403	775,210

a Only Mammalia class and Reduviidae family.

**Table 2:** WorldClim bioclimatic variable description

	No. of bioclimatic variables	Levels
WorldClim actual 1.4	19	10
WorldClim future model HadGEM2‐AO 1.4	19	10

**Table 3:** Number of grid cells by resolution

Resolution	MX	MX–US
8 km	107,776	378,176
16 km	26,944	94,544
32 km	6,736	23,636
64 km	1,684	5,909

SPECIES’ base operation is to count co‐occurrences between variables—a computationally expensive task. The direct data representation for a set of *n* variables on a *w* × *h* grid is a *w* × *h* × *n* Boolean matrix of presences which being both large and sparse is unmanageable and inefficient. In SPECIES 1.0, we use two data representations, the first using an incidence array assigned to each spatial cell and the second being the dual of this cell‐centered representation, where a set of geometry‐id arrays are associated with each variable, with each geometry‐id pointing to an element of the space partition where the variable is present.

The SNIB data model, and corresponding curated taxonomical tree, is the basis for the point‐collection data model. Point distribution ingestion is a two‐step process: First, the system filters out all points that are not within the valid region; second, for each variable, we locate the grid cells that contain at least one point of the variable's distribution and associate the corresponding set of cells. The variable is then added to the database.

Due to SPECIES’ requirement of bringing all spatial data to the same resolution, raster datasets are discretized into categorical spatial distributions. In this step, an ordinal categorization onto *N* quantiles (in the current version *N* is fixed to 10 and corresponds to the deciles) is computed for all variables in order to generate an equal area discretization of the continuous data. The categorized raster is then projected onto *N* indicator Boolean rasters.

### 3.2 Architecture

SPECIES’ architecture is modular and composed of three separate components: a database builder, a data processing engine, and a web client with a GUI. The database is currently built from two main data sources: species record data and raster data. Some structure is assumed on the record data, existence of taxonomical information, record date, geographical information (longitude and latitude), and a URL associated with each record. From this data, a series of SQL scripts transform the data and construct the database. Raster data are first manipulated into discrete form with Python and then integrated into the PostgreSQL DB.

**Figure 1:**
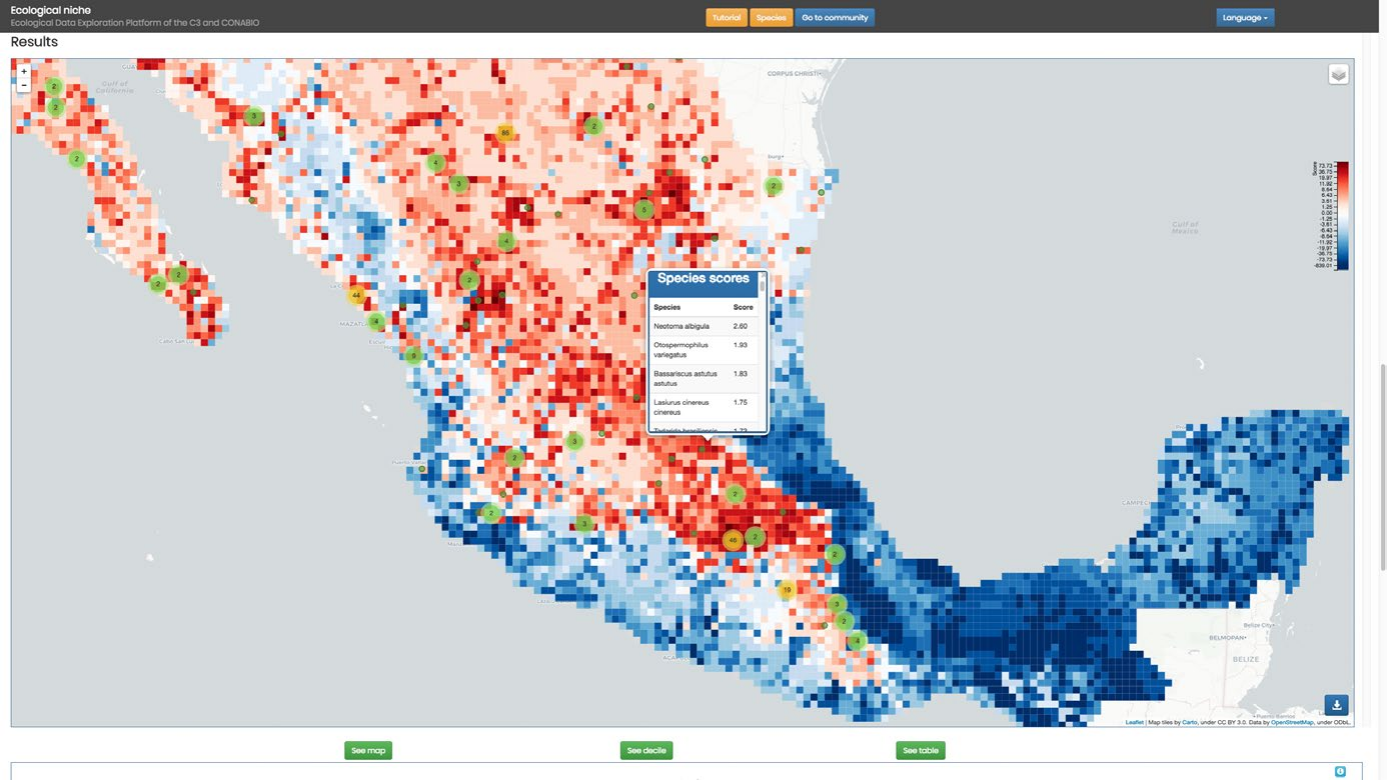
The user can explore detailed information associated with each cell

The GUI and visualization client were created with standard web technologies: HMTL5, JavaScript, and CSS3. Additionally, for interactive maps and visualizations, we use Leaflet and D3 (Data Driven Documents). The GUI is composed of two principal parts: niche and community. In the niche map, as seen in Figure 1, data for the target taxon are displayed for each cell, allowing for a review of the principal niche contributions there.

**Figure 2:**
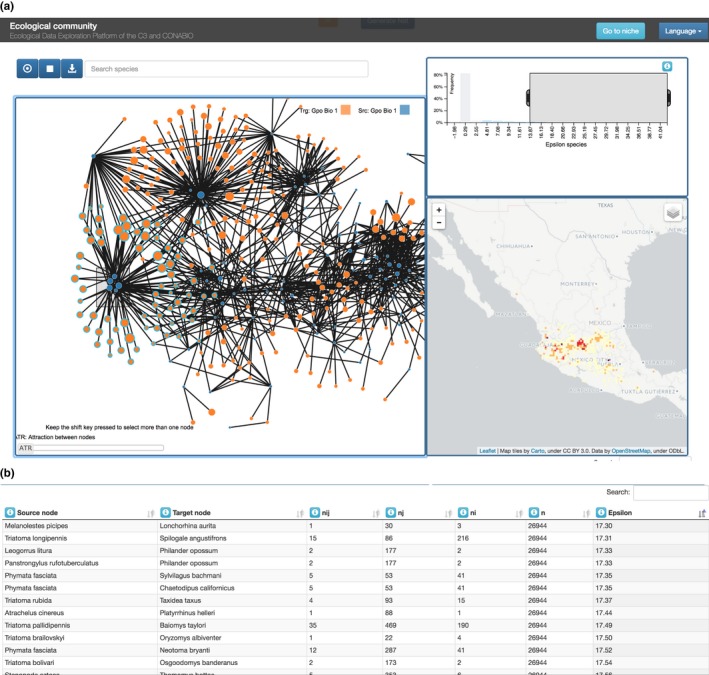
Different views of the spatial correlation results. (a) ε histogram (top right), inferred network (left), heat map that shows which areas have more of the selected species (bottom right). (b) A table displays the quantities associated with each correlation (network link)

In community, there are three visualization components showing linked views of the data (Figure 2a). The main view is the inference network itself, which provides an interactive visualization, where the user can obtain information about the size of the sample for each variable represented in the network, and the geographic distributions of any subnetwork. The remaining two views are as follows: a histogram of the link weights (ε values), which works as an interactive filter for the links and generates changes to the network and the table below, and a map, which displays the variables count distribution as heatmaps.

## 4 EXAMPLES

In this section, we present two brief working examples, for a more detailed discussion please refer to the tutorial in the Supporting Information Appendix S1.

### 4.1 Ecological niche inference

As a first use case, we consider the ecological niche of the jaguar. We first select the jaguar by its scientific name, *Panthera onca*. The system then displays relevant information about the species.

**Figure 3:**
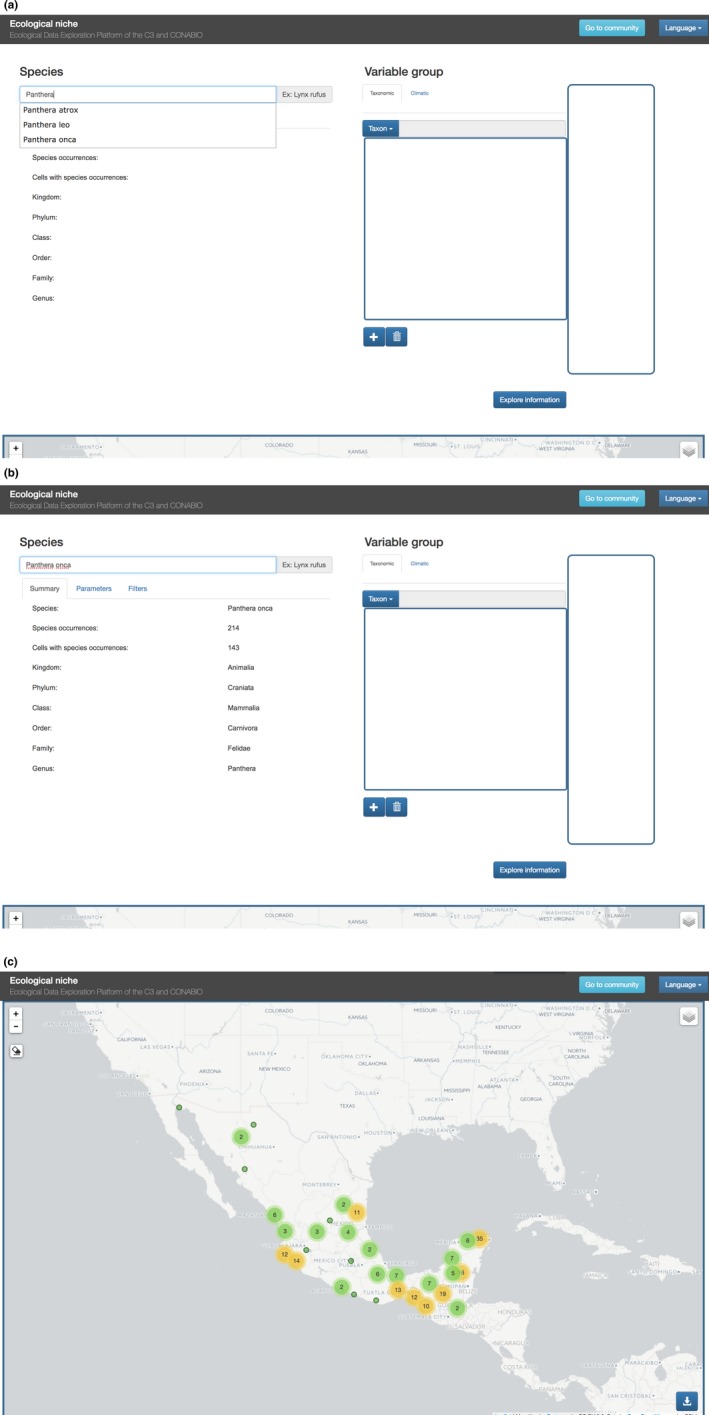
Initial setup screens (a, b) and resulting map (c). (a) Initial screen. (b) Jaguar data summary. (c) Jaguar observations distribution

**Figure 4:**
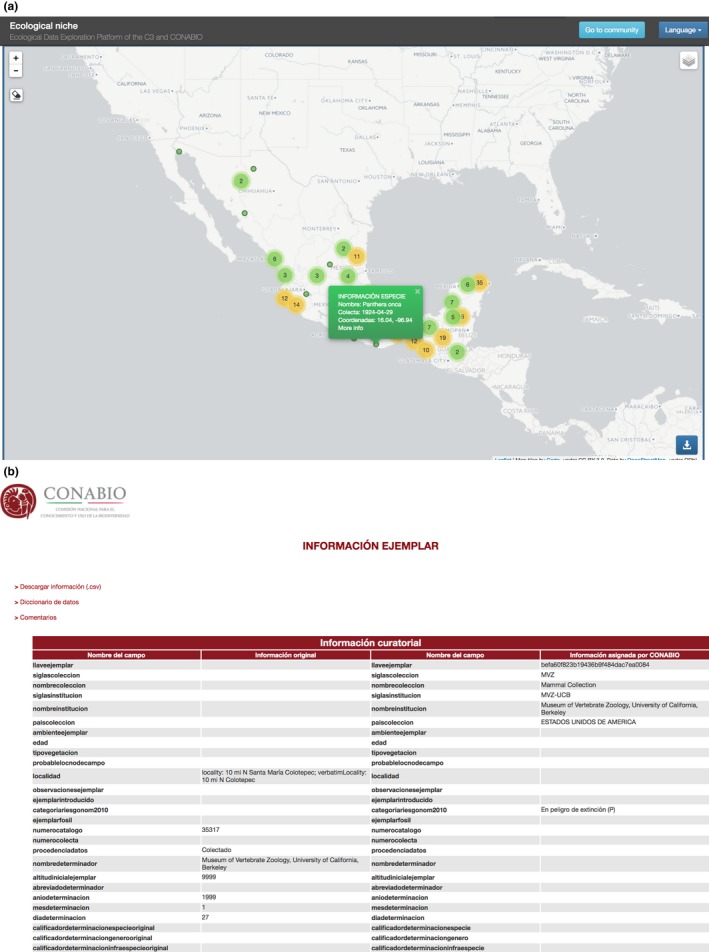
(a) Observation info box. (b) Observation's meta‐data from source database

In the corresponding map (Figure 3c), the system displays the collection points for the chosen species. Clicking on a point yields the meta‐data for that register (Figure 4a). If we decide a particular observation is not appropriate, we can remove it by clicking on the eraser icon and then clicking on the point to be removed.

**Figure 5:**
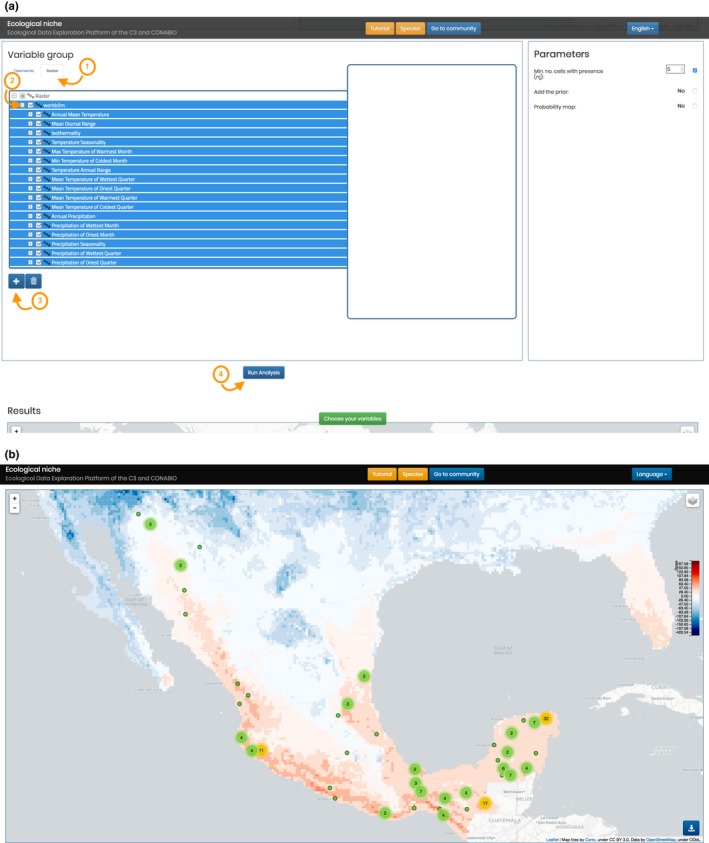
(a) Add the abiotic variables group and run the analysis. (b) Niche model for Jaguar using WorldClim variables

We can then iteratively choose the covariate groups for the niche model. For example, we may first choose only abiotic variables (Figure 5a), generating the corresponding potential distribution map (Figure 5b). We may then add a group of biotic variables—for example, the mammals in the database—and rerun the analysis. Even high dimensional analyses with many covariates run quickly thanks to the efficiency of the Naïve Bayes algorithm and SPECIES’ data model.

The subsequent distribution map is only one element of the information provided by SPECIES. Below the map, there is a bar graph that summarizes information about the niche scores of the grid cells by deciles, with decile ten showing the average score for the 10% of highest scoring cells, that is, those cells the most favorable conditions, while decile one contains the lowest scoring cells, that is, those cells which represent the least favorable condition. The graph also disaggregates the total score average by the chosen groups of variables, showing the average by group.

The deciles graph is interactive, each bar acting as a filter for the table just below. If the user clicks on a bar, the table lists the variables present in the corresponding decile. In this way, the user can examine which covariates are most associated with the niche of the target species and those which are most “antiniche.” Each row in this table corresponds to a variable and contains its name, followed by its ε and score values, and the percentage of cells in the decile that feature the variable.

**Figure 6:**
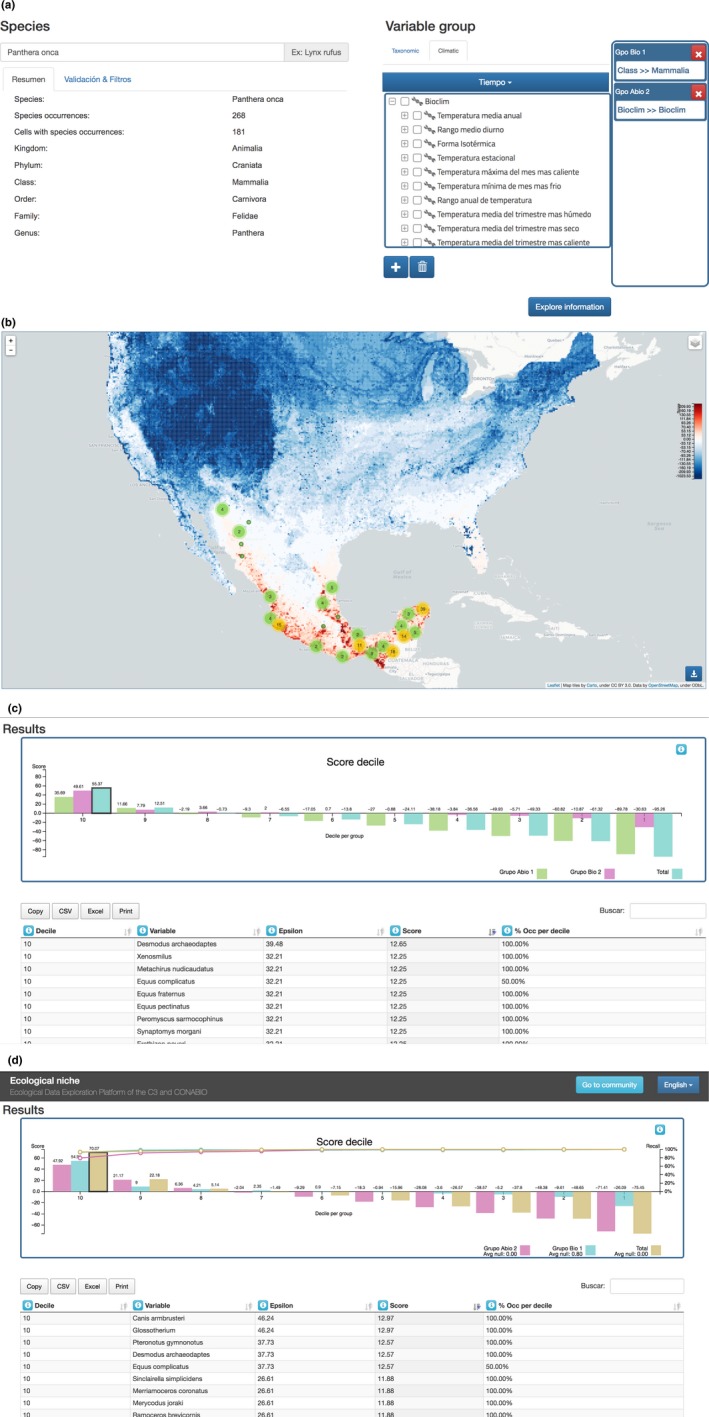
(a) Configuration to infer ecological niche for the Jaguar with validation. (b) Potential SDM for Jaguar using WorldClim and all species of Mammals in the databases. (c) Covariates sensibility deciles. (d) Per decile average recall (fivefold cross‐validation) of the Jaguar distribution model

When the user enables the *validation* option in the initial setup, the system displays a curve over the bar graph which shows the average of the cumulative proportion of presences in each decile averaged over the five iterations (Figure 6d). This curve represents the recall of the model and is a measure of its predictive power. By comparing the performance of the abiotic and biotic variables used in jaguar's niche model in terms of recall, we can see that the biotic covariates choses lead to a model that is more predictive than the abiotic one. In this context, this means that the chosen biotic covariates are more predictive than the abiotic ones in determining those optimal niche regions associated with the highest probability to find the jaguar. However, when we generate a model that integrates both covariate types, the model performance increases even further.

An important advantage of the SPECIES platform is that it allows us to establish an ecological profile for each individual cell or decile, variable by variable, thereby allowing us to quantify the relative influence of each niche variable at each geographic location and its impact on the presence of a species there. As the top decile of scores best characterizes the ideal niche, we may construct a list of the most relevant abiotic and biotic variables for this decile and rank them from highest to lowest in terms of ε values, or of score values. Considering the potential importance of predator–prey interactions between *P. onca* and other mammals, one would expect that the availability of food resources, and any corresponding change, should be an important factor in determining its range. Reviewing the species in the top decile we note that, of 18 confirmed jaguar preys distributed in Mexico (Aranda, 1994; Ávila‐Nájera, Palomares, Chávez, Tigar, & Mendoza, 2018; Chávez, Ceballos, & Amín, 2007), 15 are in the top decile, while 12 (80% of preys) are in the top 20 highest ranked species by ε. In summary, a model that includes different covariate types not only leads to more accurate prediction models, but also leads to a much fuller and more comprehensive understanding of the species niche.

Thus, the species list ranked by ε (high to low values) serves as a predictive model, with the hypothesis that the highest ranked species correspond to those species most likely to exhibit a biotic interaction, without any pretense to stating that there definitely exists a biotic interaction. Given that co‐occurrence is a necessary but not sufficient condition for an interaction, such a statistical association does not prove that there is a direct “causal” interaction between these taxa. However, such inferences can be checked and confirmed against current knowledge, as with the jaguar preys, or may be used to construct new hypotheses that may be then checked empirically.

The jaguar example may be repeated for any number of species in the SNIB. For instance, selecting the butterfly species *Baronia brevicornis* and building a model with biotic covariates comprising the plant genus *Acacia* (with 85 species), one notes that the highest ε corresponds to *Acacia cochliacantha*, which is its unique food resource (Soberón & Townsend Peterson, 2005). On the other hand, selecting a columnar cactus species (*Pachycereus pringlei*) and building a model with biotic covariates comprising 853 bird species we note that the fifth species in the ranked list is a woodpecker (*Colaptes chrysoides*), a bird species that, at first glance, one would not normally expect to be associated with cacti. However, reviewing the literature, we found that this woodpecker nests mainly in this species thereby establishing a nontrophic biotic relationship (Zwartjes & Nordell, 1998). Both these further examples show that SPECIES is a tool that may allow us to infer and test potential biotic relationships, once again emphasizing that its role it to generate hypotheses about such relationships that must be tested against known results or by generating new ones.

### 4.2 Ecological network inference

**Figure 7:**
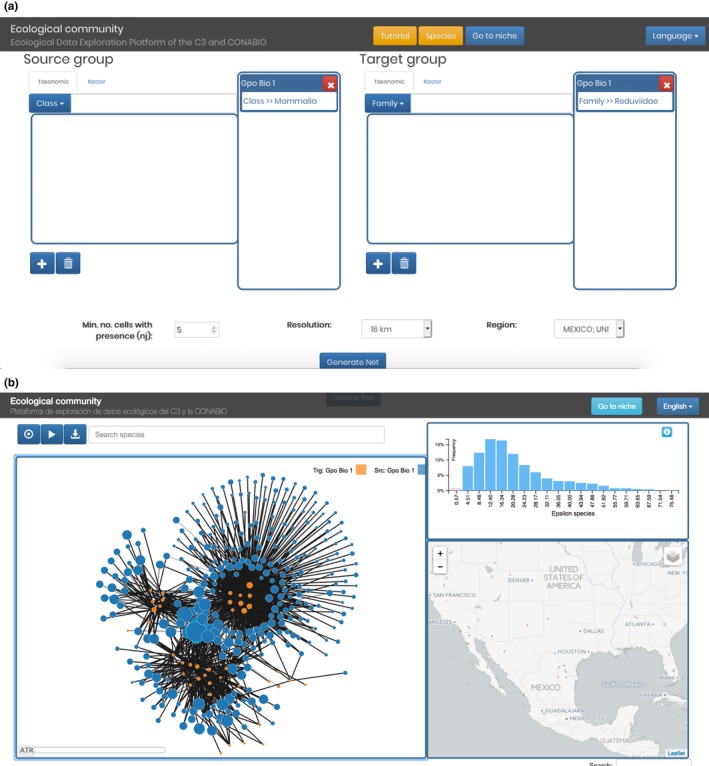
(a) Mammalia versus Reduviidae network inference setup. (b) Infered network of Mammalia vs Reduviidae species

The other modality of SPECIES is at the community level. The user defines groups of target variables and groups of source variables. For example, the class Mammalia may be the source group, and Reduviidae (which includes the genus Triatoma, known vector of Chagas disease) may be the target group. Figure 7 shows the corresponding setup and the resulting network. In the network visualization, we see three windows: The first (left of the image) is the network graph itself, the second (top right) is the histogram of correlations, and the remaining window (bottom right) is the richness heat map.

The network graph is an interactive visualization. The color intensity of the edges is proportional to the significance of the correlation between the nodes it connects, while node size is proportional to the number of grid cells occupied by the variable. On the search bar above the network, the user can type a name and the nodes that match the name will be highlighted. When the user points to a node, an info‐box pops up with the name of the variable and its number of occurrences. If the user clicks on the node the map on the right will then display the known distribution of that variable, and the neighbors of the node will be highlighted.

The data associated with the network may be downloaded as a CSV file which can then be included in any third party network analysis tool.

As with the niche interpretation, the inferred relations inherent in the network are not sufficient to unambiguously identify biotic interaction. However, the network does offer a visualization of the structure of statistical dependencies between our target nodes which, by exploring different network metrics we may be led to discover species pattern associations which, in turn, may inform us about potential biotic relationships. In our example, we selected Chagas vectors (triatomine species) and potential mammal hosts. The resulting network comprised 22 triatomine species and 390 mammals. We first identified which vector species were most connected (i.e., highest network degree), as these represent the vectors with the greatest potential to infect multiple hosts, such as *Triatoma dimidiata* (239 links). Furthermore, another nine vector species are linked to more than 100 mammals, and these species represent a high risk of Chagas transmission.

On the other hand, we can also check the mammal network degree focusing on confirmed hosts of Chagas disease (36 species; Rengifo‐Correa et al., 2017), of which 58% have a network degree <10 links, and which should be candidates for surveillance plans. Dividing the species list ranked by ε into quartiles, we found that 12% of confirmed host species are in the top quartile and that this percentage decreases toward the lower quartiles, showing that the ranked list provides a general model for predicting the most important potential hosts for Chagas disease.

## 5 FUTURE WORK

### 5.1 More data

Given the multi‐factorial nature of ecological niches, an important goal is to include more data of both different types/formats and from different geographical regions. In particular, to include in data from large international datasets such as GBIF. Although data issues can lead to erroneous conclusions, the SPECIES platform itself facilitates the detection of anomalous points and/or data which can then be checked by hand.

Another important area is the inclusion of more bespoke datasets from individual researchers or groups. As the philosophy of SPECIES is to integrate data from distinct data sources within a central modeling platform, rather than distribute a software to potential users, we plan to form a community of users such that the inclusion of their data can also leverage the large public datasets already present.

Given the capacity of SPECIES to use data associated with different time intervals, we also plan to emphasize and develop SPECIES’ capacity to model and predict dynamical changes in distributions, niches, and communities. Presently, this functionality is restricted to the inclusion of future climatic rasters. Another important future avenue of work is the inclusion of much richer meta‐data, associated with biotic variables other than purely taxonomic labels.

### 5.2 Methodology

A challenge for the SPECIES platform when using large numbers of predictor variables is how to identify, disentangle and, most importantly, interpret any observed statistically significant relationships. For example, to what degree a significant correlation is associated with a known, or plausible, “direct” interaction, such as in parasitism or predation, versus a known or plausible “indirect” interaction, such as the relation between a carnivore and a plant that is a principle food source of one of its prey species. Presently, we take a pragmatic approach, identifying an “interaction” as any variable that contributes to the presence probability of a given species. Such hypotheses can be tested by more detailed empirical analysis, as has been done in the case of the interaction between disease vectors and disease hosts (Stephens et al., 2016). Additionally, there is the important question of how to disentangle confounding factors. For instance, to determine whether a potential biotic relation is confounded by a shared climate preference. The methodology behind the SPECIES platform can be used to estimate the degree of confounding (Stephens, Sánchez‐Cordero, & González Salazar, 2017). The next version of SPECIES will include this functionality.

### 5.3 Open source project

SPECIES is an open‐access web application, with an open‐access API. Although this philosophy has been present since its inception, there remain several improvements to make in the near future in terms of documentation and code organization before welcoming and interacting in an organized environment with a potentially large community of contributors.

## 6 CONCLUSIONS

SPECIES is a powerful, flexible, easy to use analysis platform, based on a well‐founded methodology and with descriptive output. It is a system that allows for the exploration and incorporation of ecological data—both abiotic and biotic—of arbitrary spatio‐temporal resolution, and its subsequent integration into predictive models for both ecological niche and geographic distribution, while allowing for a direct comparison of the relative contributions of different variable types. It also provides an ecosystemic, community‐based analysis for inferring relations between different biota, such as the relation between disease vectors and potential disease hosts. An important philosophical difference between SPECIES and other modeling systems is that it is based on using large, existing datasets as a basis for modeling. The motivation for this is that ecological niches and communities are much more multi‐factorial than can be comprehended via small, bespoke datasets with relatively few variables. The data model behind SPECIES is optimized to carry out such high dimensional computations on the fly in order to support fast hypothesis prototyping and testing.

## CONFLICT OF INTEREST

None declared.

## AUTHOR CONTRIBUTIONS

C.R.S. and R.S.‐A. conceived the development of SPECIES. C.R.S. and C.G.‐S led implementation of the methodology. R.S.‐A. led software development. C.G.‐S wrote use cases and tutorial. E.R., J.M.B., and J.C.S. developed the backend of SPECIES. J.C.S. developed the GUI and visualization tools. C.R.S. and R.S.‐A. led writing of the article. All authors helped in testing the software and contributed critically to the draft and gave final approval for publication.

## Data Availability

All data used in SPECIES 1.0 are publicly available at the following URLs: SNIB: GBIF.org (23 April 2018) GBIF Occurrence Download https://doi.org/10.15468/dl.ikfkok
Mammals USA: GBIF.org (08 March 2017) GBIF Occurrence Download https://doi.org/10.15468/dl.99cphq
WorldClim 1.4: University of California, Berkeley and Robert Hijmans. 2004. WorldClim high resolution global climate surfaces. Knowledge Network for Biocomplexity. https://doi.org/10.5063/aa/knb.165.2.

## ENDNOTES

1 We use presence/no presence as the principle point‐collection datasets available in SPECIES are presence only.

2 Recall = true positives/(true positives + false negatives).
