# Supplementary material for: SPECIES: A platform for the exploration of ecological data
Source: Ecol Evol. 2019 Jan 28;9(4):1638–53. doi: 10.1002/ece3.4800 (PMC6392378; doi:10.1002/ece3.4800)
Supplement: Supplementary file 1 [file ECE3-9-1638-s001.pdf]

SPECIES 1.0

Constantino González-Salazar, Christopher R. Stephens, Raúl Sierra Alcocer, Juan Carlos Salazar Carrillo, Juan Barrios Vargas, Everardo Robredo and Enrique del Callejo Canal

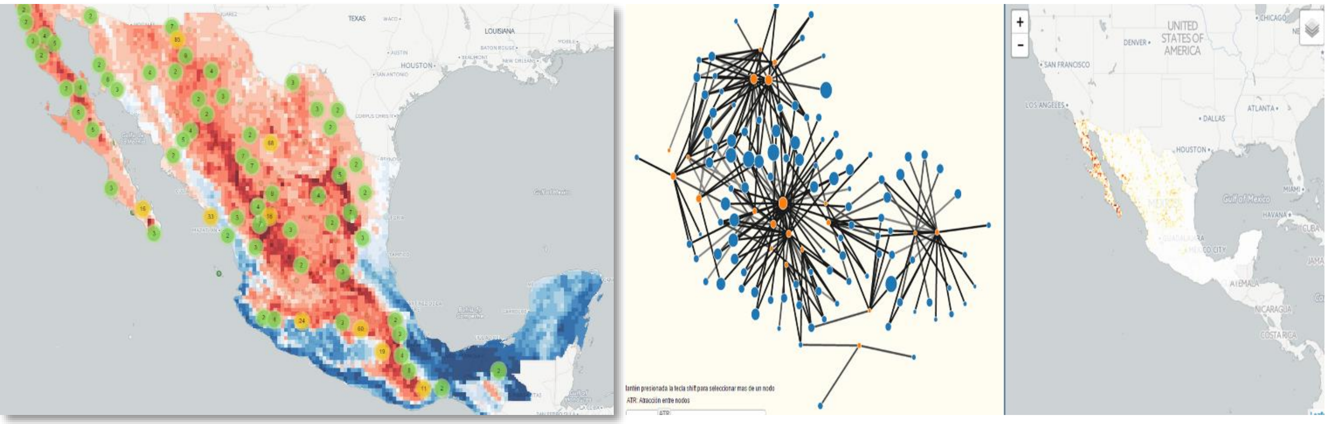

This tutorial is a basic introduction to use the web platform SPECIES, which is an interactive tool for the analysis of ecological niches and forecast species potential distribution, as well as to build Complex Inference Networks to identify potential species interactions. SPECIES development is supported by C3-Centro de Ciencias de la Complejidad, Universidad Nacional Autónoma de México and the Comisión Nacional para el Conocimiento y Uso de la Biodiversidad (CONABIO).

SPECIES 1.0

A brief tutorial

Currently, it has been an important increase of digital data of biodiversity and environmental information, such as museum specimen collection data, climatic and topographic raster layers. Furthermore, with the creation of open databases like the Global Biodiversity Information Facility (GBIF) (www.gbif.org), or WorldClim - Global Climate Data (www.worldclim.org), there are many data that are now publicly available. The challenge is to find tools with which to transform that data into knowledge, in ways that are useful for a wider range of users.

Here, we give a basic introduction of SPECIES, which is a computer tool for the exploration and analysis of geographic data to constructed species ecological niches, species potential distribution and Complex Inference Networks for community analysis. SPECIES uses a spatial data mining framework, and takes as input data any spatial variable (e.g. collections points of any mammal species or temperature values) from a pre-defined geographical region, identifying statistical associations based on the degree of co-occurrence between our target variables, for instance species-climate, species-habitat or species-species associations.

In order to determine a co-occurrence between geographic variables SPECIES uses a uniform rectangular grid that divided the region of interest into regular spatial cells, x_α, and then counted co-occurrences within each x_α for a class, C, and a subset of potential predictive variables, $\boldsymbol{X}$. To quantify which spatial variable co-distributions show a statistically significant correlation, relative to the null hypothesis that their distributions are independent and randomly distributed over the study region, SPECIES calculate the statistical diagnostic epsilon, $\varepsilon(C|X)$, where values of $|\varepsilon| > 1.96$ correspond to a greater than 95% confidence that the co-occurrences occur at a rate inconsistent with the null hypothesis. Epsilon is building block form which species distributions, species niches and complex inference networks can be constructed. The basic hypothesis is that ecological interactions can be inferred from the relative distributions of spatial variables.

For a broadly explanation of spatial data mining method implemented in SPECIES and description of the data used, see:

Christopher R. Stephens, Raúl Sierra-Alcocer, Constantino González-Salazar, Juan Barrios Vargas, Juan Carlos Salazar Carrillo, Everardo Robredo and Enrique del Callejo Canal. **SPECIES: A Platform for the Exploration of Ecological Data**.

SYSTEM DESCRIPTION

Conversely to many ecological modelling applications which are downloadable software, SPECIES uses a web-based application, and it can be accessed using web browsers, like Google Chrome, Microsoft Edge, Mozilla Firefox or Safari, from URL http://species.conabio.gob.mx/. SPECIES provides tools to share the results of an analysis: tables can be exported in CSV or Excel format; maps can be downloaded in vector format (shapefiles); and ecological networks as CSV files. Another way to share an analysis is by sharing the setup of the analysis via a URL that reproduces the exact setup of the experiment

Additionally, SPECIES is currently linked to National Biodiversity Information System (SNIB for its initials in Spanish) of the CONABIO, therefore, the users have access to a main database of Mexican biodiversity that includes around 8 million of georeferenced localities of 81,603 species of flora and fauna. SPECIES also includes 19 bioclimatic variables from WorldClim data base.

SPECIES have two modules of analysis: 1) ecological niches and 2) ecological community. To show the workflow in SPECIES, we presented two study cases for each module of platform http://species.conabio.gob.mx/:

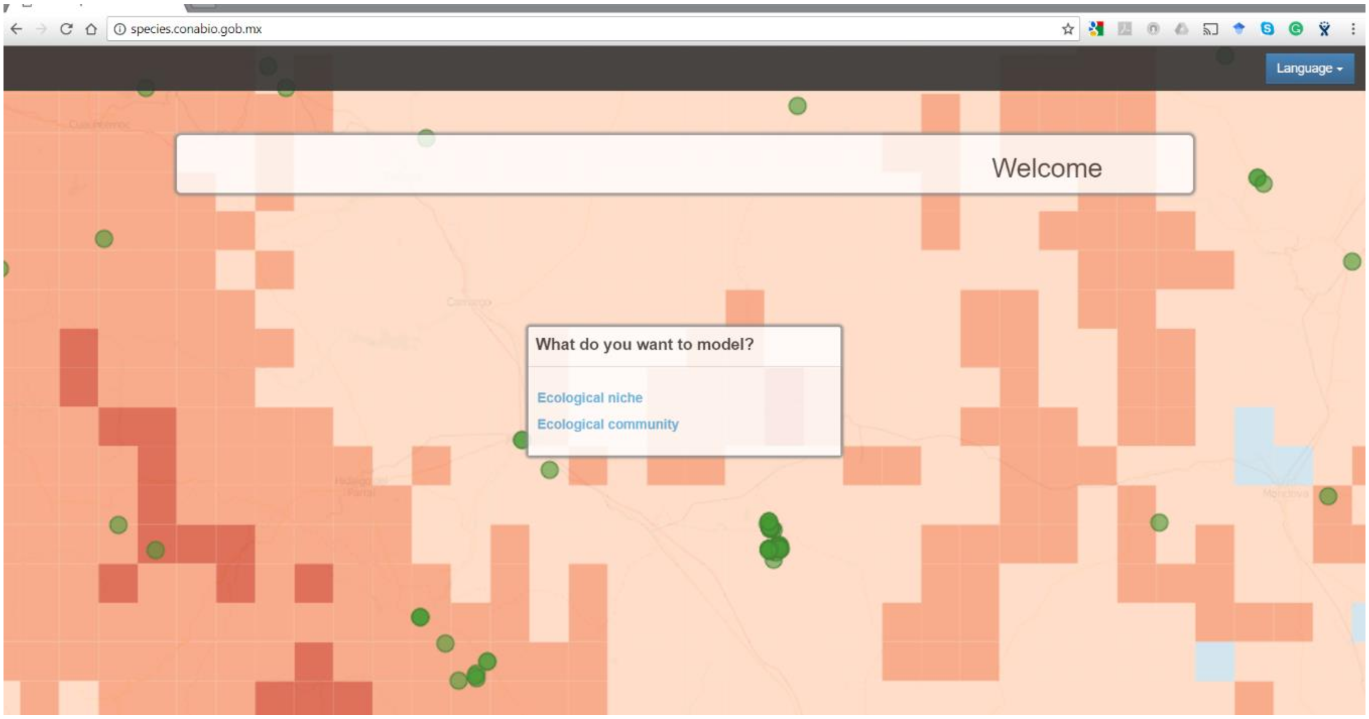

ECOLOGICAL NICHE MODULE:

Study case*: Integrating biotic and abiotic variables to evaluate potential distribution of Lynx rufus in Mexico*

An important goal in niche modelling is to determine and compare the contribution of biotic and abiotic niche features, to obtain a better understanding of their relative importance in species distribution. Therefore, a niche modelling methodology that allows us to include different types of variables, such as climate and biotic interactions, offers a fruitful framework within which to explain the ecological processes that occur from local to regional scales, and understanding which factors, barriers or biotic interactions are important for a particular species in a particular geographical location. In other words, to better understand the relation between the geographical distribution of the species and its niche.

We showed SPECIES workflow for analysis of ecological niche and potential distribution of bobcat (*Lynx rufus*), integrating biotic and abiotic predictors. Because of *L. rufus* is a carnivore species we used other mammal species (potential preys) as biotic predictors:

1. <u>Accessing the module "Ecological Niche"</u>

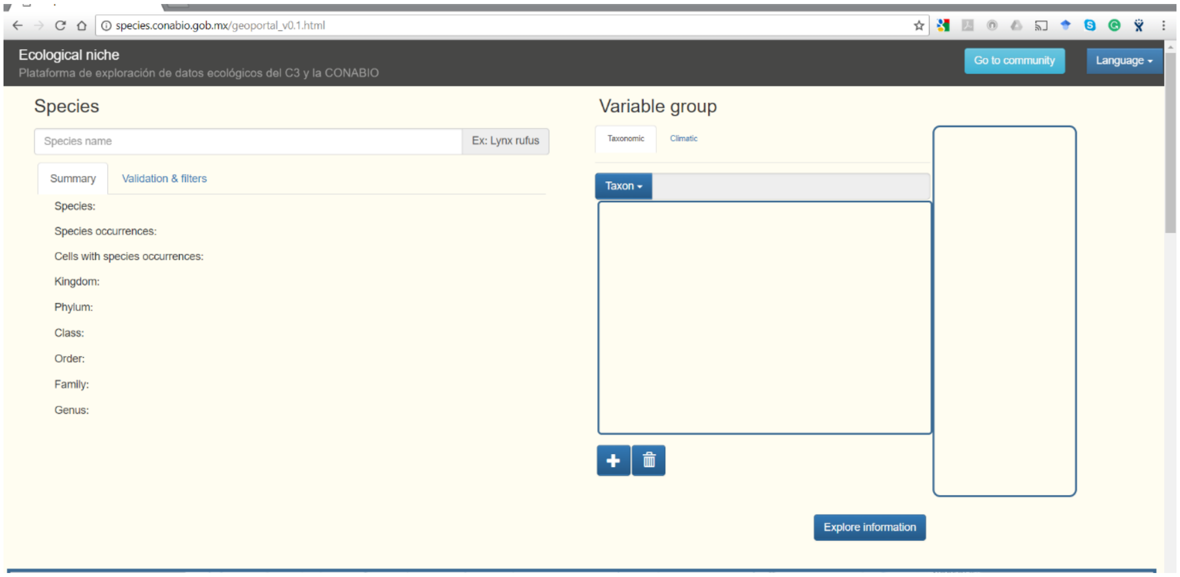

This first screen shows us two sections: 1) “Species”, where we selected our target species, and 2) “Variables group”, where we selected our predictor variables.

2. <u>Selecting input data</u>

We write species scientific name, i.e. *Lynx rufus* to selected its geographic data

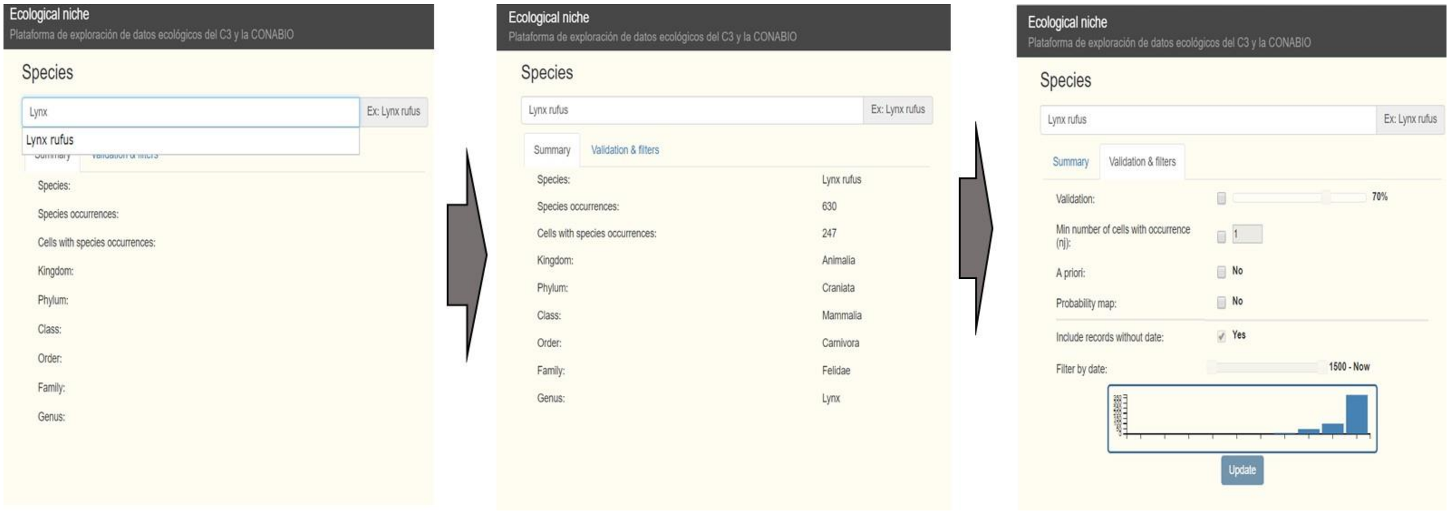

The system displays information about the species, in the "Summary" tab, we find taxonomic information, number of collections points (bobcat has 630 records), and the number of cells with species occurrences (for a grid of 20km^2 bobcat has 247 unique cells). In "Validation & filters" tab, we can selected that SPECIES performed a validation analysis, (default option is 70% of cells for training and 30% cells for testing), minimum number of cells with occurrences, (i.e. the minimum number of unique cells that any predictor variables should have for the analysis), a probability map to display species potential distribution, and a filter by dates, which allows us to selected a particular period of time.

The system display species localities in a map, where we can explore this information. By selecting any collection point we can access to the metadata of that record. If we identified an incorrect record, we can remove it by clicking on the eraser icon (upper left corner) and then clicking on the point to be removed

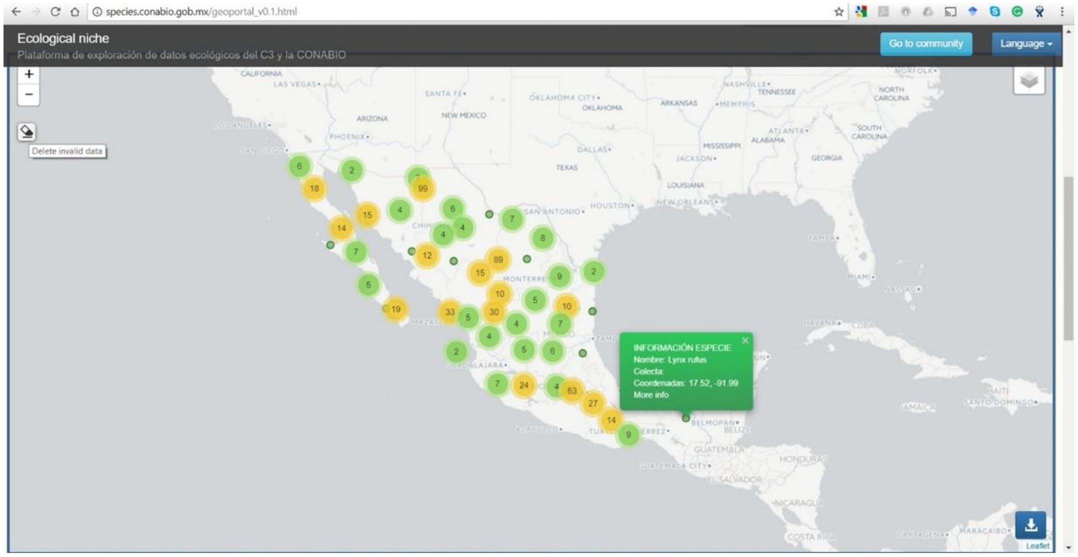

The next step is to select our predictor variables. In "Variables group" SPECIES has, at the moment, two types of variables: 1) biotic variables, which are collection points of around 80,000 flora and fauna species, and 2) abiotic variables, including 19 bioclimatic layers.

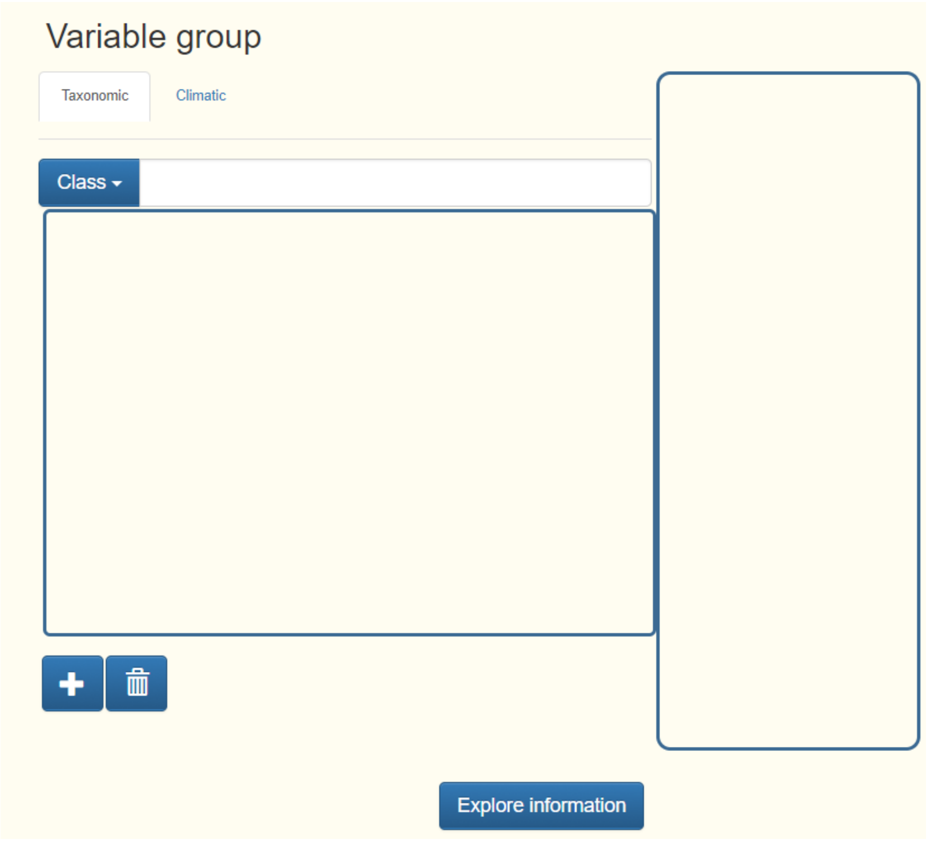

The system allows us to select biotic variables at any taxonomic level. In our particular case, due to *L. rufus* is a carnivore species, we selected Class Mammalia (1), to which potential bobcat's pyres belong. Then, we selected those groups within the Class Mammalia that we want to include (2). Finally, we add these groups for the analysis (3). Thus, we have biotic predictors to characterize bobcat´s ecological niche.

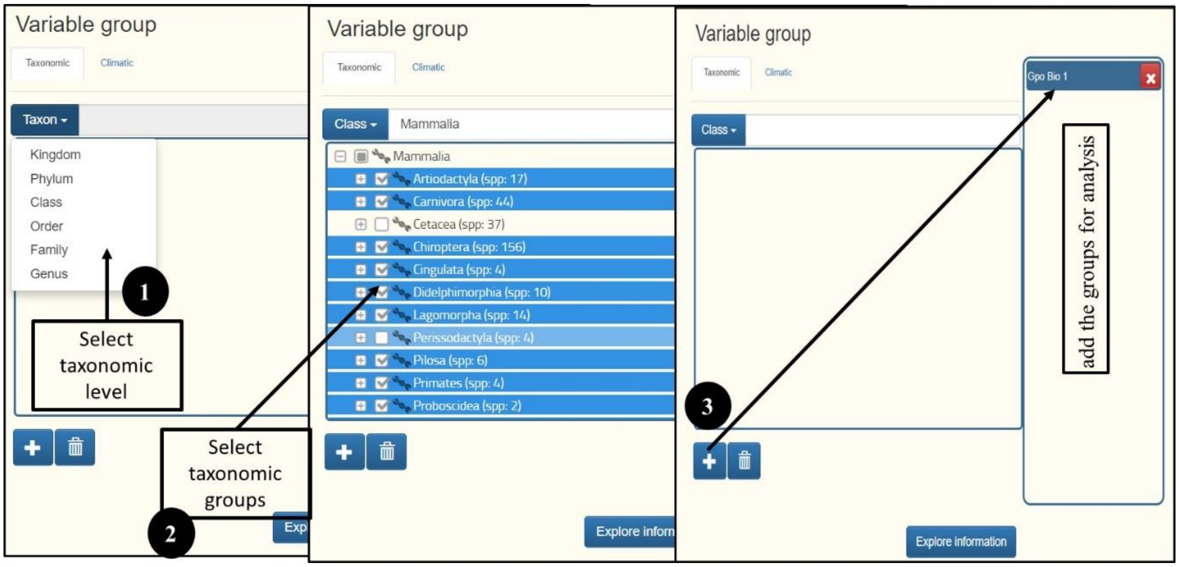

In the next tab we can select climatic variables. Users can select the 19 bioclimatic layers or chose only those that are relevant for your particular study. In this case we added the 19 variables.

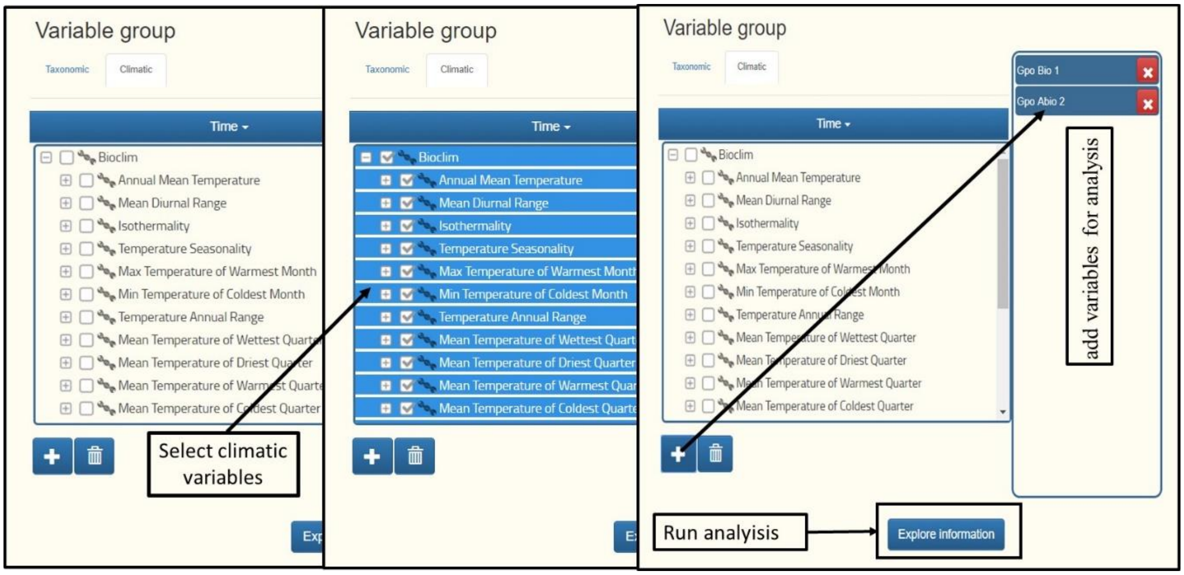

Finally, we run the experiment with "Explore information" button.

3. Exploring Ecological Niche module Outputs

A first result is a potential species distribution map, which is built by a Naïve Bayes approach calculating score contribution for each predictor variable. A completed explanation of how geographic map is constructed can be seen in the manuscript of SPECIES (Stephens et al). In summary, this map showed a gradient form optimal niche conditions (dark red colour) to suboptimal niche conditions (dark blue colour) determined by a combination of biotic and abiotic factors. Dark red areas represent those regions with high probability for *L. rufus* to be present.

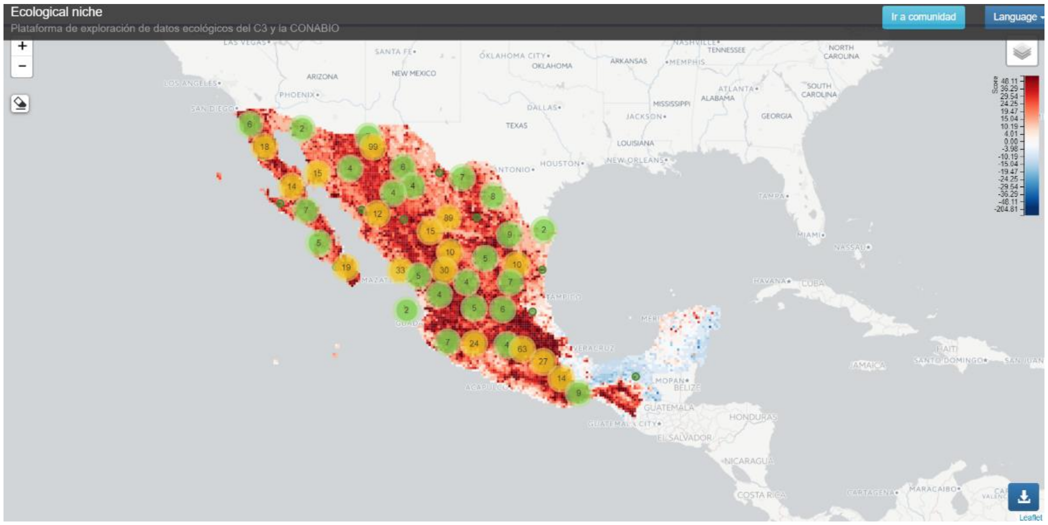

If we select any cell, the system display the information contained in that cell, shows us each variable contributions (Scores values) for the potential presence or not presence of our target species.

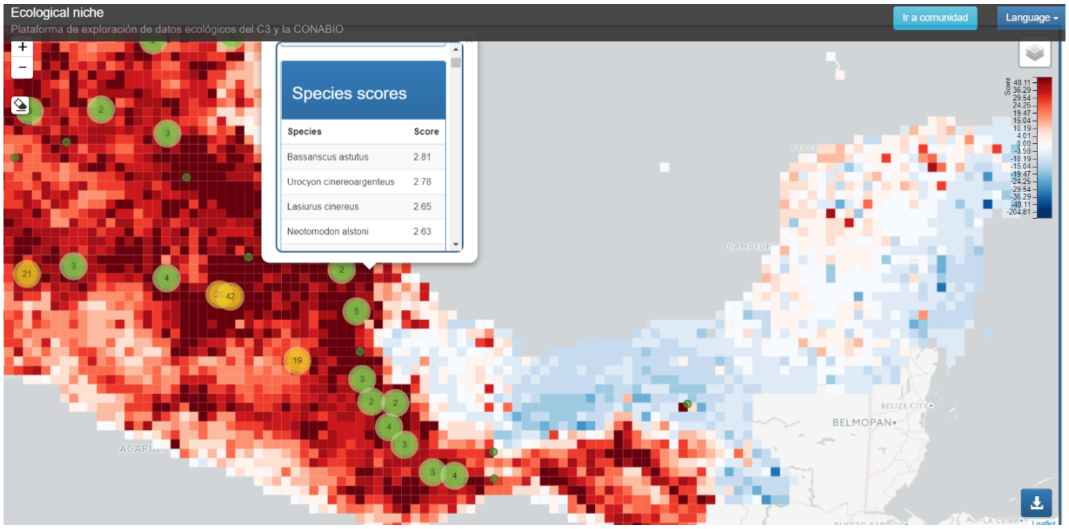

This map can be downloaded clicking in the icon . The information is sending to your email. Map is in vector format (*.shp) to be displayed in any software of geographic information system, e.g. QGIS, ArcGis, Diva-Gis.

To examine model performance as a function of score values, SPECIES divided the grid cells into deciles. The 10th decile corresponds to the 10% of grid cells with the highest score values, the 9th decile to the next 10% of grid cells with highest score values, etc. This allows us to establish predictability profiles across the different score deciles. A performance measure used here is to calculate for each score decile the percentage of associated target species collections. The larger the percentage in the higher decile is, the better, more discriminating model. A random model would yield 10% of true positives in each decile whereas a perfect model would locate all point collections in the highest ranked cells. Thus, large changes in score passing from one decile to another correspond to the fact that the associated model discriminates well between one decile and another.

The system shows a decile bar graph, where bars represented average score by decile for each type of variable individually and combined (e.g. biotic and abiotic). The deciles graph is interactive, each bar acting as a filter for the table just below. If the user clicks on a bar, the table lists the variables present in the corresponding decile. Each row in this table corresponds to a variable and contains its name, followed by its ε and score values, and the percentage of cells in the decile that each feature occupy. By using ε we can determine for each decile what the most correlated niche factors are. This table can be exported in CSV or EXCEL format, or copy directly. When the user enables validation in the initial setup, the system displays a curves over the bar graph, which showed the cumulative proportion of presences predicted correctly in each decile. The system calculate curves for each type of variable selected and for the combination of them, allowing us to note which variable or combination, is more predictive.

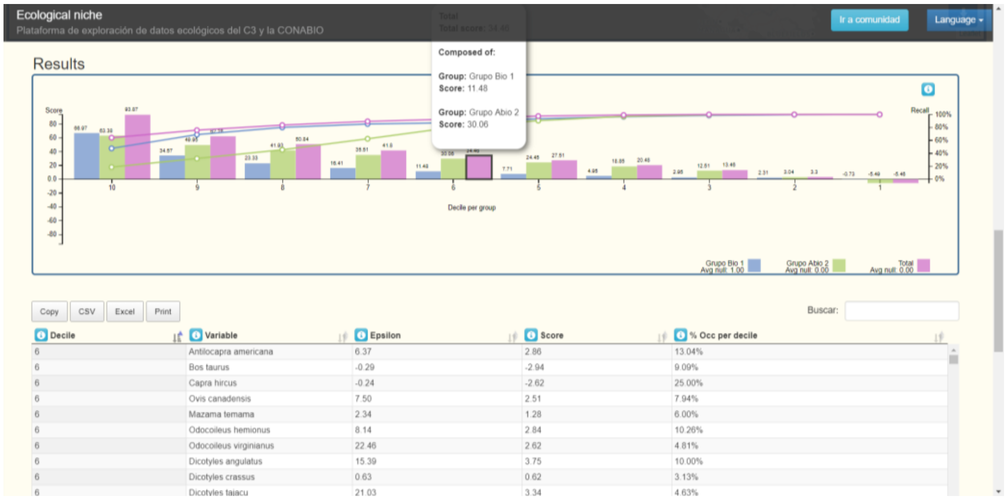

To the end of web page, the system displays a table with all variables used in the analysis, it contains variable name, number of cells where co-occur with our target species (n_{ij}), number of cells of variable (n_j), number of cells of our target variable, (n_i) (i.e. cells with *L. rufus* presence), total of cells (n), ε and score values, and taxonomic classification for species. This table can be exported in CSV or EXCEL format, or copy directly.

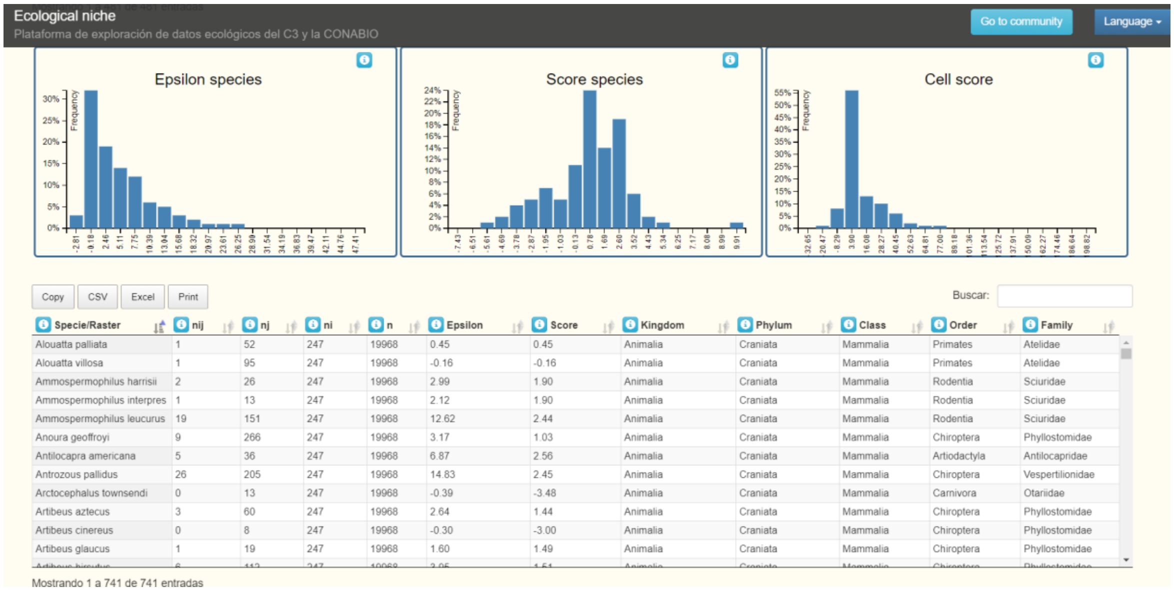

Specie/Raster	nij	nj	ni	n	Epsilon	Score	Kingdom	Phylum	Class	Order	Family
Alouatta palliata	1	52	247	19968	0.45	0.45	Animalia	Craniata	Mammalia	Primates	Atelidae
Alouatta villosa	1	95	247	19968	-0.16	-0.16	Animalia	Craniata	Mammalia	Primates	Atelidae
Ammospermophilus harrisii	2	26	247	19968	2.99	1.90	Animalia	Craniata	Mammalia	Rodentia	Sciuridae
Ammospermophilus interpres	1	13	247	19968	2.12	1.90	Animalia	Craniata	Mammalia	Rodentia	Sciuridae
Ammospermophilus leucurus	19	151	247	19968	12.62	2.44	Animalia	Craniata	Mammalia	Rodentia	Sciuridae
Anoura geoffroyi	9	266	247	19968	3.17	1.03	Animalia	Craniata	Mammalia	Chiroptera	Phyllostomidae
Antilocapra americana	5	36	247	19968	6.87	2.56	Animalia	Craniata	Mammalia	Artiodactyla	Antilocapridae
Antrozous pallidus	26	205	247	19968	14.83	2.45	Animalia	Craniata	Mammalia	Chiroptera	Vespertilionidae
Arctocephalus townsendi	0	13	247	19968	-0.39	-3.48	Animalia	Craniata	Mammalia	Carnivora	Otariidae
Artibeus aztecus	3	60	247	19968	2.64	1.44	Animalia	Craniata	Mammalia	Chiroptera	Phyllostomidae
Artibeus cinereus	0	8	247	19968	-0.30	-3.00	Animalia	Craniata	Mammalia	Chiroptera	Phyllostomidae
Artibeus glaucus	1	19	247	19968	1.60	1.49	Animalia	Craniata	Mammalia	Chiroptera	Phyllostomidae

The work-flow of module "ecological niche" explained here is based on a previously published manuscript of this particular study case. For an extensive demonstration of analysis that it can be performed with SPECIES outputs, you can see: González-Salazar, C., Stephens, C. R. & Marquet, P. A. (2013). Comparing the relative contributions of biotic and abiotic factors as mediators of species' distributions. Ecol. Modell. 248, 57–70. Certainly, users are able to perform study cases using just one type of variable, biotic or abiotic.

COMMUNTY MODULE:

Study case: *Using Complex inference Networks for prediction in emerging diseases: study case Leishmaniosis*

Emerging zoonoses are an important global threat to public health. Distinct to non-zoonotic diseases, their transmission cycle involves complex ecological interactions. Host range, in particular, is a crucial factor in determining disease risk and the potential for adequate interventions. Of course, transmission cycles can potentially depend on a huge number of factors, both abiotic and biotic. For instance, for the transmission of Leishmaniasis, the parasite generically requires the presence of two wild hosts – a mammal reservoir and an insect vector – to complete its life cycle. Unfortunately, little is known about these ecological components of the disease – reservoirs and vectors and their mutual interactions. Consequently, there is a need to develop methodologies that can predict which organisms could be important reservoirs and vectors for a particular disease.

Complex inference networks have been an important tool for prediction in zoonoes, here, we presented a work-flow of SPECIES to build networks to infer potential vector-host interactions for Leishmaisis disease selecting mammal species as potential hots, and species of genus *Lutzomyia* as vectors.

1. Accessing the module "Community"

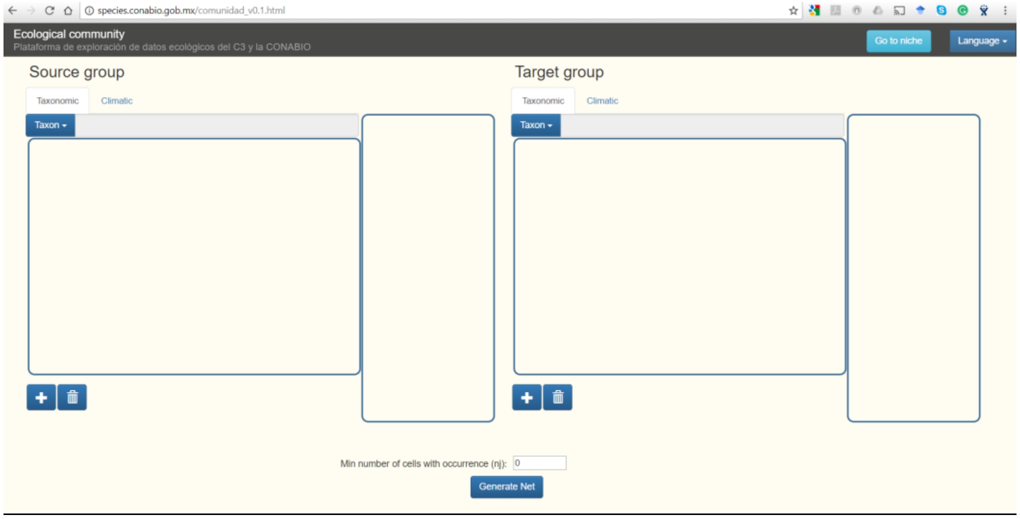

This first screen shows us two sections: 1) “Source group”, where we selected our species of interest, and 2) “Target group”, where we selected species with which we are interested to identify potential interactions. Additionally, the platform give us the option to select the minimum number of cells with occurrence for any species.

1. Selecting input data

We can selected our *source group* at different taxonomic levels. In our particular case, we selected genus *Lutzomyia* (1). Then, we selected species belonging this genus (2). Finally, we add these species for the analysis (3). Thus, we have potential vectors of Leishmaniasis

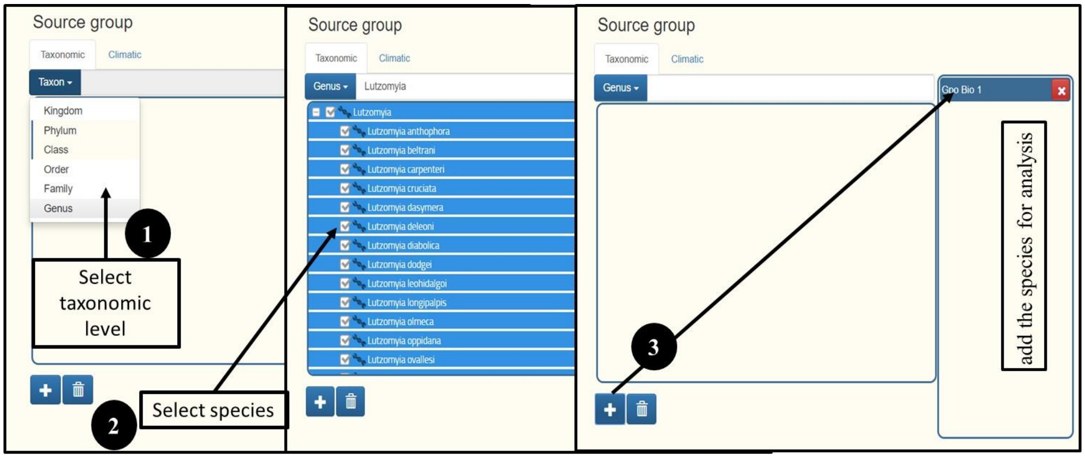

To select our "Target group" we should carry out similar steps, first we chose taxonomic level (1), in our case we selected Class: Mammalia. Then, we selected which Orders, Families Genera or Species we will use for our analysis (2), in this case we selected Orders Chiroptera and Rodentia. Finally we add these species for the analysis (3). Thus, we will build a complex inference network, where nodes are *Lutomyias* and Mammal, and strength of links between them are determined by epsilon values (ε).

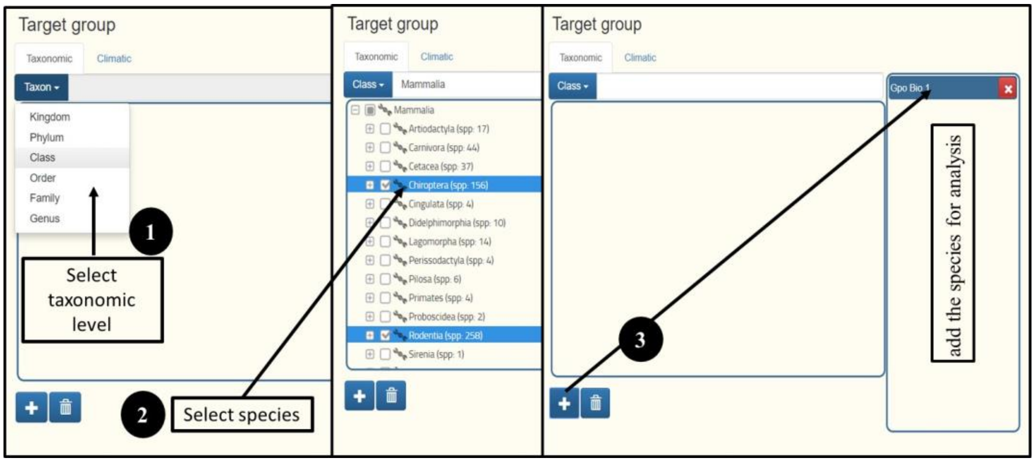

We choose species with minimum five cells with occurrences and we run the analysis clicking on button, 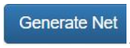

.

3. Exploring Community module Outputs

To explore community outputs we see three windows. In the first (left of the image) is the network graph itself. Network show us source and target nodes with different colours and sizes. In the example, *Lutzomyia* species are in blue and mammals are in orange. Nodes size is related to the number of species occurrence, so, big nodes are species with presence in a great number of cells, and small nodes is the contrary. If users select any node, the system display the species name and number of cells with its occurrences; additionally, all nodes linked to previously selected node are lighted. When you selected a group of nodes, the map in the bottom right window shows us richness for each cell, this richness heat map allows us to identify geographic patterns of species richness. The third window (top right) is the histogram of correlations, with epsilon range values in x-axis, here, we can indicated an umbral of ε and the system jus display those pair of species connected within this ranges of values.

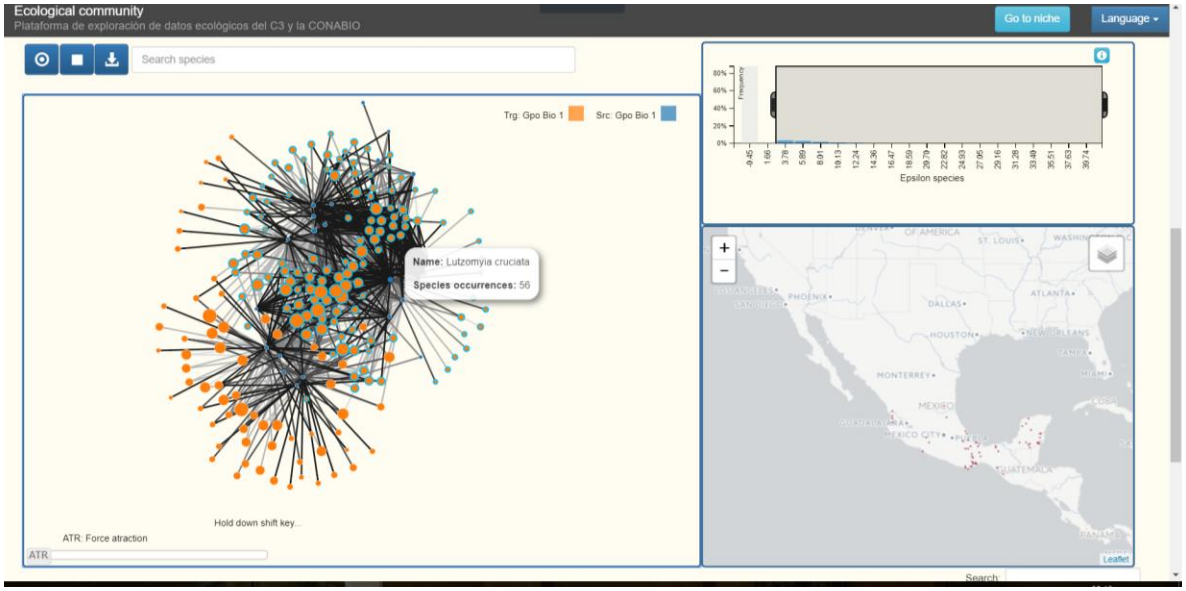

At the end of web pages, there is a table with all pair species combination for our target and source groups. This table contains names of species, number of cells where co-occur source and target species (nij), number of cells of target species (nj), number of cells of source species, (ni), total of cells (n) and ε values. When we download the network clicking in button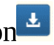, the system send us this table in CSV format, which can be opened in Excel our other software for network analysis.

Source node	Target node	nij	nj	ni	n	Epsilon
Lutzomyia anthophora	Peromyscus perfulvus	1	44	1	19968	21.25
Lutzomyia anthophora	Notocitellus adocetus	1	51	1	19968	19.73
Lutzomyia anthophora	Osgoodomys banderanus	1	173	1	19968	10.65
Lutzomyia anthophora	Oryzomys fulgens	1	196	1	19968	9.99
Lutzomyia anthophora	Lasiurus cinereus	1	220	1	19968	9.42
Lutzomyia anthophora	Sigmodon mascotensis	1	221	1	19968	9.40
Lutzomyia anthophora	Neotoma bryanti	1	287	1	19968	8.22
Lutzomyia anthophora	Balantiopteryx plicata	1	291	1	19968	8.16
Lutzomyia anthophora	Dermanura phaeotis	1	338	1	19968	7.56
Lutzomyia anthophora	Baiomys musculus	1	427	1	19968	6.69
Lutzomyia anthophora	Baiomys taylori	1	469	1	19968	6.37
Lutzomyia anthophora	Sciurus aureogaster	1	486	1	19968	6.25
[illegible]	[illegible]	[illegible]	[illegible]	[illegible]	[illegible]	[illegible]

These results allow us to infer potential vector-host interactions between *Lutzomyia* and mammals species, and can be tested subsequent by fieldwork. Originally, this complete study case was presented in Stephens, et al. (2009). Using biotic interaction networks for prediction in biodiversity and emerging diseases. PLoS One 4, e5725, and its predictive model was successfully tested by fieldwork, finding novel hosts of Leishmaniasis. (Stephens, et al. (2016) Can you judge a disease host by the company it keeps? Predicting disease hosts and their relative importance: A Case Study for Leishmaniasis. PLoS Negl. Trop. Dis. 10(10), e0005004.

SPECIES gives the option to use climatic variables in networks analysis, so, future studies can be used this approach to explore and analysis climatic affinities of species, as well as similarities and differences in climatic preferences among a group of species.
